# A Nutrition Report Card on food environments for children and youth: 5 years of experience from Canada

**DOI:** 10.1017/S1368980020000130

**Published:** 2020-08

**Authors:** Alexa R Ferdinands, Dana Lee Olstad, Krista M Milford, Katerina Maximova, Candace IJ Nykiforuk, Kim D Raine

**Affiliations:** 1 School of Public Health, 3-300 Edmonton Clinic Health Academy, University of Alberta, Edmonton, AB T6G 1C9, Canada; 2 Department of Community Health Sciences, Cumming School of Medicine, University of Calgary, Calgary, AB, Canada

**Keywords:** Monitoring, Nutrition policy, Report Card, Food environment, Children

## Abstract

**Objective:**

In 2014, a Nutrition Report Card (NRC) was developed as a sustainable, low-cost framework to assess the healthfulness of children’s food environments and highlight action to support healthy eating. We summarise our experiences in producing, disseminating, evaluating and refining an annual NRC in a Canadian province from 2015 to 2019.

**Design:**

To produce the NRC, children’s food environment indicator data are collected, analyzed and compiled for consensus grading by an Expert Working Group of researchers and practitioners. Knowledge translation activities are tailored annually to the needs of target audiences: researchers, practitioners, policymakers and the public. Evaluation of reach is conducted through diverse strategies, including tracking media coverage and website traffic. Assessment of impact on diets and health outcomes is planned.

**Setting:**

Alberta, Canada.

**Participants:**

Not applicable.

**Discussion:**

The grading process has facilitated refining the NRC to enhance its relevance and utility as a tool for its target audiences. Its public release consistently captures media interest and policymakers’ attention. The importance of partnerships in revealing data sources and in strategising to enhance policy approaches to improve food environments is apparent. The NRC has benchmarked progress and stimulated dialogue regarding healthy food environments for children.

**Conclusions:**

The NRC may help to foster a supportive climate for improving the quality of children’s food environments. As an engaging and accessible document, the NRC represents a key mechanism for collating data related to children’s food environments and ensuring it reaches the audiences best positioned to use it. Efforts are underway to expand the NRC across Canada.

Food environments shape the availability, affordability and social acceptability of food ‘choices’^(1)^. Ready access to energy-dense, nutrient-poor foods facilitated by current food environments may be contributing to the high prevalence of unhealthy eating behaviours among children and youth in Canada^(2,3)^. This is concerning because childhood represents a critical period for establishing healthy eating behaviours^(4)^. Additionally, diet-related chronic diseases are one of the greatest contributors to premature mortality in Canada^(5)^.

Given the tendency for unhealthy eating behaviours and determinants of these behaviours (e.g., poverty) to persist across the life course^(4,6–8)^, prompt action is required to ensure that children’s food environments support, rather than undermine, their health. Benchmarking and publicising government tobacco control initiatives have previously helped to generate support for stronger government actions and policies to reduce tobacco consumption^(9)^. Similar measures might be leveraged to incite action to improve childhood nutrition.

In 2005, a Report Card on Physical Activity for Children and Youth was created to evaluate Canadian progress in improving children’s physical activity behaviours and environments^(10–12)^. Annual Report Card release elicits substantial media and public attention and has been cited by policymakers as an evidence source during policy development^(13)^. Moreover, this Report Card is well-regarded internationally, such that in 2014, fifteen nations released their own contextualised physical activity Report Cards^(12)^.

Given the success of the physical activity Report Card, the absence of a similar mechanism to monitor and report progress in improving the quality of children’s food environments represented a missed opportunity to identify strengths and possibilities for change. In 2013, researchers, practitioners and policymakers seized a funding opportunity to form a collaborative to provide leadership and support to develop, implement and evaluate policy activities for chronic disease prevention^(14)^. As part of this initiative, the team oversaw the conceptualisation^(15)^ of a framework for Alberta’s Nutrition Report Card on Food Environments for Children and Youth, hereafter abbreviated as the ‘NRC’ (https://abpolicycoalitionforprevention.ca/evidence/albertas-nutrition-report-card/). The goals of the NRC are ‘to *monitor* the state of children’s food environments and supportive policies*, inform* stakeholders of the state of these environments and policies*, engage* society in a national discussion, and outline a policy-relevant research agenda for further *study’*^(15, p. 287)^. In this way, the collaborative aimed to advance a solutions-oriented agenda related to childhood health promotion. Target stakeholder groups of the NRC include researchers, practitioners, policymakers and the public.

Development of the NRC framework, including its key objectives and theoretical underpinnings, has been previously described^(15)^. Briefly, the NRC framework includes five environment domains identified as influencing children’s eating behaviours^(16)^: physical, communication, social, economic and political (Table 1). Indicators and benchmarks are subsumed within these five food environments. *Indicators* are key areas where it is important to take action to improve children’s eating behaviours. *Benchmarks* are specific targets for each indicator that may help to improve children’s eating behaviours, if they are met.

**Table 1:** The five food environments^(16,17)^

Environments	Description
Micro-environments	
Physical	The physical environment refers to what is available in a variety of food outlets, including restaurants, supermarkets, schools, worksites, as well as community, sports and arts venues
Communication	The communication environment refers to food-related messages that may influence children’s eating behaviours. This environment includes food marketing as well as the availability of point-of-purchase information in food retail settings, such as nutrition labels and nutrition education
Economic	The economic environment refers to financial influences, such as manufacturing, distribution and retailing, which primarily relates to cost of food. Costs are often determined by market forces; however, public health interventions such as monetary incentives and disincentives in the form of taxes, pricing policies and subsidies, financial support for health promotion programmes and healthy food purchasing policies and practices through sponsorship can affect food choices
Social	The social environment refers to the attitudes, beliefs and values of a community or society. It also refers to the culture, ethos or climate of a setting. This environment includes the health promoting behaviours of role models, values placed on nutrition in an organisation or by individuals and the relationships between members of a shared setting (e.g., equal treatment, social responsibility)
Macro-environment	
Political	The political environment refers to a broader context, which can provide supportive infrastructure for policies and actions within micro-environments

Building on this foundation, the objectives of this article are to describe how our aforementioned collaborative applied this framework^(15)^ to produce five annual NRC (2015–2019) for the Canadian province of Alberta. Specifically, we reflect on NRC: (i) production, (ii) knowledge translation activities, (iii) refinements, (iv) successes and challenges, (v) lessons learned and (vi) future directions.

## Design: Nutrition Report Card production

Several stepwise procedures are undertaken to produce the NRC each year. These procedures require the involvement of many key players, including the Expert Working Group (EWG).

### The Expert Working Group

The EWG is currently comprised of thirteen researchers and practitioners working in diverse fields (nutrition, education, recreation, law and public health) in different provinces (Alberta, Ontario and Québec) across Canada. Most EWG members have participated in NRC production from its inception, although there has been some flux in membership along the way. In addition to grading NRC indicators and helping to craft themes and recommendations, the EWG is engaged sporadically throughout the year for purposes such as reviewing data collected to date to ensure that key sources of information are not overlooked and in facilitating data access.

### The production process

**Fig. 1:**
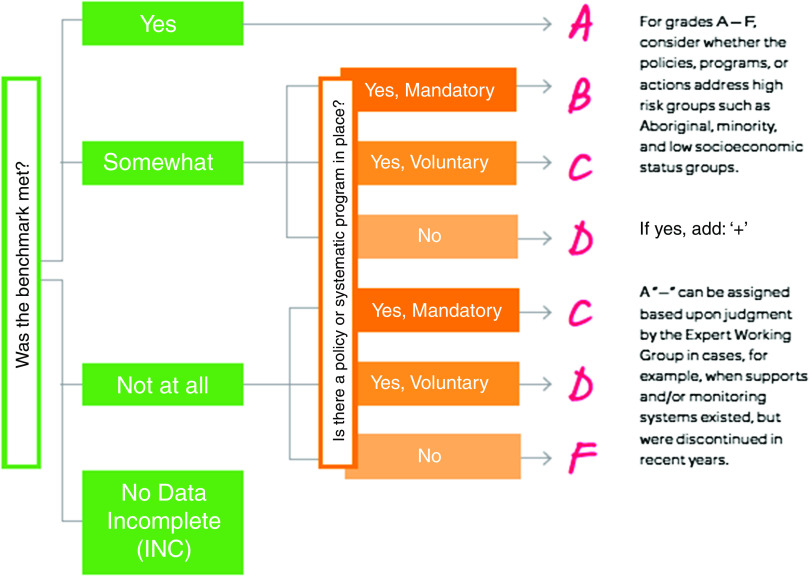
The Nutrition Report Card grading process^(17)^

Annual NRC production involves the following steps: data collection and analysis, individual grading, grading consensus process, calculation of final grades, development of recommendations and compilation of a final report. Graduate and undergraduate students contribute to year-round data collection, using general web searches; keyword literature searches; government, institutional and non-governmental organisation websites; policy databases; contacting key informants by telephone and email; and networking with established health organisations. A stepwise grading process (Fig. 1) guides experts through assigning grades for each indicator^(15,17)^. Grades of A through F are assigned that reflect achievement of, supports for and monitoring of indicator-specific benchmarks. Each grader assesses the indicators individually based upon a summary data document. Individual grades are synthesised into a single document, and graders then convene in a full-day consensus meeting to reach agreement on the grades and discuss potential recommendations for action. The grading process serves to highlight current strengths within Alberta’s food environments and areas for improvement, with unmet benchmarks serving as a call to action. After grading, research, practice and policy recommendations are created for each indicator. Final products include a full-length written report (with detailed explanations of grades and underlying data), a summary report and an infographic (started in 2017). Although the first NRC was published in the month of January, subsequent NRC were and continue to be published annually in September, capitalising on the increased attention paid to children’s wellness at the start of the school year.

### Key informants and partnerships

Building upon the EWG’s social networks, we established a list of key informants and their respective organisations from whom we collect data and share final reports with annually. Examples of such partnerships include those with research groups at other Canadian universities, provincial governments and non-profit organisations. In some instances, the data shared have already been analyzed, while in others, we are provided with raw data requiring secondary analysis. For example, environmental health inspectors provide us with lists of all food outlets in the province, which we then code based on their healthfulness to assess the quality of physical food environments. In turn, gathering these data led us to consider additional analytic approaches for understanding that data, such as expanding beyond the NRC indicators to explore competitive food environments surrounding children’s activity settings. Illustrations of key partnerships that played an integral role in NRC production are outlined below:

- *Alberta Policy Coalition for Chronic Disease Prevention*^(18)^. The Coalition is a collaborative consisting of seventeen organisations that have come together to coordinate efforts, generate evidence and advocate for policy change to reduce chronic disease in Alberta. The collaborative conducts evidence syntheses on NRC-related topics, such as nutrition labelling and sugar-sweetened beverage taxation, which support our annual literature updates concerning NRC indicators. The Coalition also plays an important knowledge translation role, drawing from NRC recommendations when advocating for policy changes to promote healthy food environments.
- *Alberta Health Services*^(19)^ is the provincial health authority. Connections with Alberta Health Services facilitate access to key data required for grading. In addition, as a result of EWG collaboration with this health authority, there has been movement towards incorporating the NRC as a tool to support provincial dietetic practice. In 2018, an Alberta Health Services webinar showed how public health dietitians could use the NRC to enhance their work, thereby expanding the scope of the NRC to health practitioners and promoting its sustainability.
- *Alberta Healthy Schools Community Wellness Fund*^(20)^. The Wellness Fund supports projects that enhance children’s health and wellness to create healthy school communities. Acquiring school-based nutrition data for the NRC can be challenging, but EWG relationships with the Wellness Fund have facilitated inclusion of nutrition-related questions into their annual school health survey, responses to which were then used to inform NRC indicators.

Evidently, these partnerships were fundamental not just to NRC production but to knowledge translation as well.

## Knowledge translation activities

Knowledge translation activities include (i) disseminating the NRC; (ii) conducting media and public awareness activities and (iii) knowledge exchange through consultations and symposia. We fulfill these activities through the following:

- *Disseminating the NRC.* A proactive dissemination strategy is constructed each year in partnership with communications experts available in house to maximise reach and impact. Our distribution list expands annually as we become aware of additional stakeholders and knowledge users. In 2017, we began delivering communication toolkits to stakeholders and knowledge users (e.g., Heart and Stroke Foundation, Dietitians of Canada), including the key informants described earlier, to accompany NRC release. This mobilisation toolkit assists partners to extend the research of the NRC and facilitates its use. Toolkit contents include key messages, quotes, prepared social media posts, an infographic, an email script, a newsletter article and a media release. Through our dissemination efforts over the years, NRC reach has spread around the globe, having been downloaded online in a total of sixteen countries to date.
- *Conducting media and public awareness activities.* Our public relations and media strategy engage local and regional media contacts (e.g., television, radio). Public release of the NRC consistently garners significant media attention, although there has been some flux over the years. Media and public awareness activities are evaluated by assessing media coverage and website traffic (Table 2). There are many possible reasons for the variability in these statistics over time, such as the fact that there are now fewer reporters to send media releases to than in past years. For example, in 2015 there were three dedicated health reporters in major Edmonton, Alberta media outlets, while today, there is only one. Additionally, communications strategies, such as methods of pitching media releases, have been modified over the years alongside changes in personnel. A communications expert introduced to the team in 2017 chose to curate a different distribution list for each media release considering a reporter’s interest and relevance of the NRC to the media outlet’s audience, rather than distributing the NRC to all potential media outlets. Although the 2017, 2018 and 2019 reports were sent to fewer contacts, the average click/open rate (an indicator of interest in the story topic) for supporting materials (the report, infographic and media release) was higher. That said, it is also likely that the NRC has become more known over time, contributing to higher click rates and downloads of the NRC.
- *Knowledge exchange through consultations and symposia*.
  - *Consultations.* With each release, we email letters about NRC findings and their relevance to various ministries (agriculture, health, education, children’s services, municipal affairs, community and social services, transportation) within the Alberta government. Over the past 5 years, we have sent out thirty-two letters and received twelve response letters from ministers indicating their interest in the NRC. Additionally, these letters have resulted in five invitations to consult with government officials.
  - *Symposia.* To date, our team has delivered more than forty presentations about the NRC in four different countries to a wide variety of audiences, including academics, practitioners, government, non-profit community organisations and parents. The purpose of these presentations has ranged from academic keynotes to building local capacity to use NRC data to foster community food environment change. These presentations have consistently been well-received, demonstrated by requests for follow-up presentations and invitations for collaborations, such as a current multi-province collaboration that has applied for funding to expand the NRC to multiple jurisdictions in Canada.

**Table 2:** Media coverage and website traffic

Output indicator		2015	2016	2017	2018	2019
Media coverage	Media release distributed	Pitched to 131 contacts on 5 January 2016	Pitched to 120 contacts on 26 September 2016	Pitched to fifty-two contacts on 28 September 2017	Pitched to twenty-five contacts on 19 September 2018	Pitched to ten contacts on 24 September 2019
	Average click/open rate for information included in the media release	15 %	N/A*	56 %	71 %	100 %
	Request to schedule interview from news outlets	Eleven news outlets	Eleven news outlets	Seven news outlets	Four news outlets	Eight news outlets
	Unique media stories generated	Thirteen	Eleven	Eight	Four	Eight
Website traffic	Unique page views	971	N/A*	1573	1639	539
	Clicks to PDF/download link	N/A*	N/A*	322	348	N/A*

NRC, Nutrition Report Card.

Definitions: Unique page views: The number of sessions during which the specified page was viewed at least once. A unique page view is counted for each page URL + page title combination (Google Analytics).

Clicks to PDF/download link: Number of times a visitor clicked on a PDF link. Clicking the link leads to PDF copy available for download. PDF links include links to the full report, summary report, infographic and media release.

Time frame for media coverage:

For the 2015 NRC, statistics reflect the period of 5–15 January 2016.

For the 2016 NRC, statistics reflect the period of 26 September–20 October 2016.

For the 2017 NRC, statistics reflect the period of 28 September–31 October 2017.

For the 2018 NRC, statistics reflect the period of 19 September–15 October 2018.

For the 2019 NRC, statistics reflect the period of 24 September–12 November 2019.

Time frame for website traffic:

For the 2015 NRC, statistics reflect the period of 1 March 2015–1 September 2016.

For the 2017 NRC, unique page view statistics reflect the period of 1 September 2017–31 August 2018.

For the 2018 NRC, unique page view statistics reflect the period of 1 September 2018–31 August 2019.

For the 2019 NRC, unique page view statistics reflect the period of 1 September 2019–12 November 2019.

Clicks to PDF/download link statistics reflect the period of 1 September 2017–12 November 2019.

* Data are not available due to shifts in methods of evaluation over time.

## Refinements to the Nutrition Report Card framework and process

The NRC is continuously refined to enhance its relevance as a knowledge translation tool for its target audiences of researchers, practitioners, policymakers and the general public. In bringing experts around the table for discussion, the grading process has proved useful in refining indicators, benchmarks, the grading scheme and overall formatting, helping to simplify the NRC and enhance its relevance for policy impact and public understanding of the importance of healthy food environments. The NRC began with forty-two indicators and was streamlined to thirty-seven indicators in 2016. Indicators and benchmarks have been removed or added based on their topical relevance. For example, a 2015 NRC benchmark addressed concerns about the presence of artificial trans fats in commercially prepared foods. However, this issue was subsequently resolved with Canada’s ban on the use of the main source of artificial trans fats (i.e., partially hydrogenated oils) by the food industry^(21)^. With policies, regulations and monitoring systems in place, there was no rationale for further assessment; therefore, this benchmark was removed in 2016. In its place, the EWG added a benchmark concerning sodium, given the 2016 release of Canada’s Healthy Eating Strategy which targets sodium as a ‘nutrient of concern’, in addition to fat and sugar^(22)^. Through discussion with the EWG, changes have also been made to indicators and benchmarks to increase clarity of wording.

**Fig. 2:**
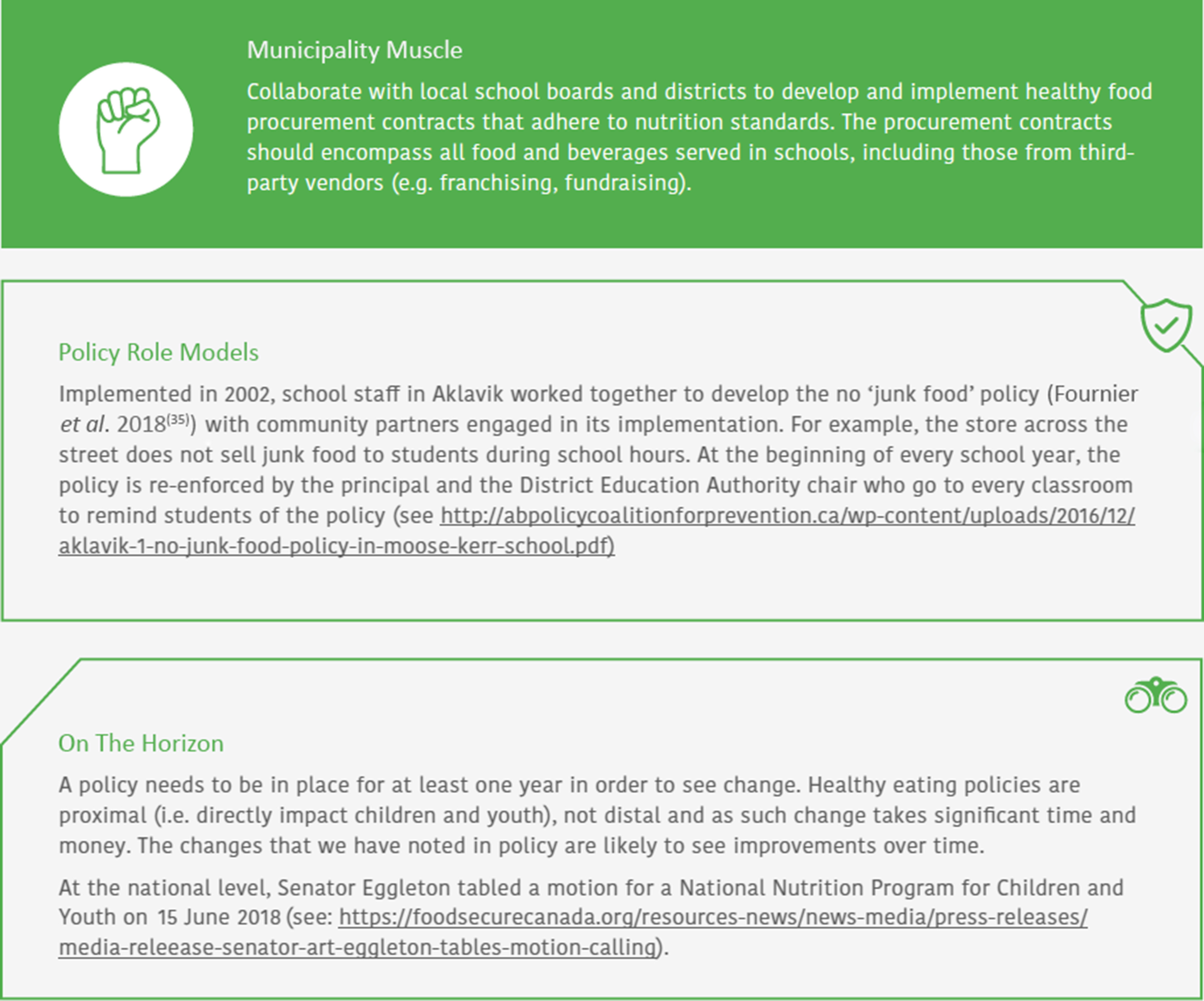
Examples of ‘municipality muscle’, ‘policy role models’ and ‘on the horizon’ highlights in relation to Indicator 1: high availability of healthy food in schools^(17, p. 20)^

To improve the utility of the NRC for the public, we have modified the visual design and content of the report over the years. In 2017, ‘on the horizon’ highlights were added to the NRC to acknowledge work in progress. For example, as observed in Fig. 2, a highlight noted the announcement of a National School Nutrition Program for Children and Youth. In the next NRC, readers could anticipate details regarding this programme’s status. These highlights may further serve as an accountability measure, prompting governmental and non-governmental organisations to follow through with their intended actions for the public good. ‘Policy role model’ sections were also added, which celebrate health champions and emphasise best practice and how-to examples that other jurisdictions can emulate, thereby building capacity. For example, Aklavik’s no ‘junk food’ policy, implemented by school staff with community partners, is featured in Fig. 2. Such stories of policy change display local communities’ strengths and can positively influence social mobilisation of resources in other communities, prompting local health promoters to similarly take action on their food environments.

Another adjustment to our knowledge translation strategy included the 2017 decision to format the NRC around a specific theme each year, providing an overarching narrative linking key findings that emerge from each subsection. This narrative, supported by multiple lines of evidence and depicted as a one-page infographic^(23–25)^, likely presents a more compelling case for action than could be achieved by, for example, publicising findings from individual studies through separate media releases over an extended time period. We have continued with this strategy since 2017. In 2017, youths’ vulnerability to unhealthy food environments emerged as an important concern within the EWG, as we observed how nutrition-related policies tended to concentrate on the health of younger children, while overlooking the needs of older youth. Hence, the theme of ‘vulnerable youths’ was featured. In 2018, the theme of ‘municipal action’ was selected, targeting municipalities for their potential to leverage social change. ‘Municipality muscle’ highlights were thus incorporated to increase municipalities’ awareness of their power and actions that they could take to improve food environments (e.g., zoning bylaws, nutrition guidelines in recreation facilities), which they may have previously overlooked. The example in Fig. 2 emphasises how municipalities can collaborate with local school boards to create healthy food procurement contracts. Finally, in 2019, we chose the theme of ‘optimal food environments for young (preschool) children’, an ongoing area of concern in Alberta. Data on childcare food environments were lacking for years prior, but in 2019, such data were released.

## Discussion: successes and challenges

These refinements have been integral to the ongoing success of the NRC. We highlight key successes, and challenges, below.

### Successes

The NRC demonstrated its ability to fulfill its aforementioned goals of (i) monitoring, (ii) informing, (iii) engaging and (iv) studying^(15)^ through the following:

- *Monitoring*. The first NRC in 2015 served as a baseline for future data collection, identifying where policy was succeeding and where work was needed to support healthy food environments. By way of NRC monitoring practices, we have tracked cultural shifts in children’s food environments in Alberta, and Canada at large, that align with NRC recommendations. For example, at the provincial level, Alberta’s School Nutrition Program^(26)^, which provides healthy meals and snacks to students, was piloted in 2016 and has since been implemented province wide. At the federal level, Canada’s Healthy Eating Strategy^(22)^, introduced in 2016, addresses the need for regulated front-of-package nutrition labelling. The NRC is valuable in regard to its ability to capture and monitor these positive changes in policy. The NRC also captures declines in the healthfulness of food environments, such as the cessation or reduction of government funding for healthy eating initiatives. In doing so, governments are held accountable to their responsibilities in promoting children’s health, given that announcements are rarely made to publicise funding cessation, leaving the public otherwise unaware.
- *Informing*. Knowledge translation activities have informed a growing list of stakeholders about the importance of investing in policy change to support positive change to children’s food environments in Canada. In its push for transparency, the NRC can help to drive policy change. Further, the NRC is supported by peer-reviewed funding, generated in a respected institution and produced by a world-renowned team of investigators, enhancing its credibility. As one means of gauging success in informing stakeholders, we conducted a stakeholder survey after the release of the 2015 and 2016 NRC. Those who downloaded the NRC were prompted to complete a pop-up survey which asked about their willingness to participate in future research. A survey, open for 2–3 months post-launch, was then emailed to those interested. Descriptive statistics were used to summarise the 2015 (*n* 20) and 2016 (*n* 14) data (Table 3). In both 2015 and 2016, most respondents came from Alberta. Respondents generally viewed the NRC as successful in terms of its ability to increase awareness of obesity prevention policies and influence policies and programmes. The majority of respondents reported believing that the NRC was successful in increasing awareness about the importance of food environments for children’s health promotion and in communicating knowledge on the current environments/policies that support or hinder healthy eating in children and youth. Most respondents also indicated that the NRC was valuable in influencing government and non-government issues and stakeholders to create and enhance policies, programmes and campaigns that improve food environments for children and youth. Finally, when asked in what ways respondents planned to use the information in the NRC, the top three uses were policy development, programme development and advocacy. Due to the low response rate, we stopped offering these surveys but were encouraged by this evidence of NRC use.
- *Engaging*. The NRC has instigated interest in creating healthy food environments at municipal, provincial and national levels, while exemplifying an innovative way to engage the public in policy action^(27)^. Our responsibility as a research team has been to advise the public and practitioners how they can adapt the NRC framework for diverse settings, where only a subset of the indicators may be relevant. The NRC also supports the work of INFORMAS (International Network for Food and Obesity/non-communicable Diseases Research, Monitoring and Action Support), which has developed a comprehensive and detailed system to monitor public and private sector policies and actions, and indicators of key aspects of the healthiness of food environments for global application at a population level^(1,28,29)^. The NRC should be regarded as complementary to this work, with its more targeted set of indicators specific to children, primary reliance on existing data and different intended audiences.
- *Studying*. Unique to the NRC is its multi-level framework, acknowledging that continuing to frame unhealthy diets as an individual problem is unproductive. Most research on diet-related chronic diseases has been at the individual level (e.g., behavioural, metabolic and genetic) or more ‘downstream’ at the population level (e.g., prevalence, school interventions), neglecting broader socio-structural factors^(30,31)^. Rather than a traditional problem-oriented approach to the study of diet-related chronic disease, the NRC is solution-oriented, in that it provides recommendations for action based on indicator grades. The NRC has also revealed its utility in identifying research gaps, whereby indicators with incomplete data for grading are highlighted as areas warranting further research.

**Table 3:** ·2015 and 2016 Nutrition Report Card (NRC) knowledge translation survey findings

Demographics	2015 NRC (%) (*n* 20)	2016 NRC (%) (*n* 14)
Jurisdiction their organisation’s mandate covers		
Alberta	50	57
British Columbia	9	7
Manitoba	0	0
New Brunswick	9	7
Newfoundland and Labrador	0	0
Nova Scotia	0	0
Ontario	9	7
Prince Edward Island	0	0
Québec	0	0
Saskatchewan	9	0
Northwest Territories	0	0
Nunavut	0	0
Yukon	0	0
Not stated	14	21
Primary role		
Clinician/health care provider	0	14
Health system worker	5	7
Researcher/evaluator	32	14
Public health practitioner/health promoter	36	29
Epidemiologist/statistician/analyst	0	7
Patient/patient advocate	5	0
Other	14	7
Not stated	9	21
Survey question	Strongly agree	Agree
Respondents who indicated that, as a result of reading the NRC, they plan to:		
Encourage their organisation to adopt a new strategy/approach	26	50
Collaborate with colleagues and/or other organisations working on the same issues	58	80
Use the same information to assist in decision making	47	70
Respondents who indicated that the information from the NRC will change their organisation’s behaviours, policies or practices	21	40
Respondents who indicated that the NRC is achieving its objective of increasing awareness about the relevance of food environments and nutrition for health promotion and obesity prevention in children and youth	94	70
Respondents who indicated that the NRC is successful in advancing and communicating knowledge on the current environments and policies that support or create barriers to improving children and youth’s dietary behaviours and body weights and that are associated with food environments and nutrition	82	80
Respondents who indicated that the NRC is valuable in influencing government and non-government issues as well as stakeholders to create and enhance policies, programmes and campaigns that improve food environment and nutrition opportunities for children and youth	59	60
	Large extent	Some extent
Respondents who indicated the percentage to which NRC information is used in their organisation to inform:		
Policy development	41	60
Programme development	41	70
Advocacy	53	70

### Challenges

NRC production has not been without challenges. These challenges pertain to:

- *Data collection*. Issues inherent to data collection include the lack and timeliness of data for some indicators. However, we hope that by identifying research gaps, data availability and quality will improve over time. Because the NRC relies largely on publicly available data, we are not always aware of relevant proprietary data.
- *Resource requirements.* NRC production requires extensive human resources and financial capital to support said resources. While the NRC was designed to be inexpensive to conduct, ongoing funding and organisational partnerships will nevertheless be required to enable annual generation and dissemination of NRC. Data collection and analyses are time intensive processes, requiring intimate knowledge of the provincial policy context to ensure adequate representation, sufficient nutritional knowledge to accurately assess the healthfulness of foods available, as well as geographic information systems expertise. Having a web-based, automatic system for nutritional assessments could help to decrease human resource requirements (e.g., an online tool like the ‘Canteen Scan’^(32)^).
- *Health inequities*. Our high-level assessments may fail to capture nuances at the local community level. For example, the NRC does not delve into the intricacies of Alberta’s multicultural landscape and the need for culturally appropriate food environments for those from different heritages. Within the NRC, it is rarely possible to address equity issues as there are limited nutrition data relating to disadvantaged groups, such as Indigenous populations, in Alberta. Because population health data in Alberta tend to be shared in aggregate form rather than by sub-populations, it becomes challenging to study inequities and discern how vulnerable populations may be differentially affected by current food environments and policies.
- *Subjectivity of grading.* Despite objective data, the grading process is fraught with value judgements, requiring discussion and debate by the EWG. For example, as part of the grading scheme (Fig. 1), a decision is made for each indicator about whether a benchmark has been met, somewhat met or not at all met. If a benchmark states that, for instance, 75 % of all foods and beverages available for sale must be healthy, a finding that 20 % of foods and beverages are healthy creates an ambiguity as to whether the benchmark has been somewhat or not at all met. The expertise of the EWG is essential for such decision making, and the consensus process helps to overcome some of this subjectivity.
- *Sensitivity of grading.* Our experiences have illuminated how the cultural and political contexts of certain jurisdictions may not necessarily align with the concept of ‘grading’. Not only is food entwined with personal and cultural values and beliefs, but jurisdictions may also be concerned about being judged or reflected upon poorly. This scenario unfolded in 2015, where another Canadian jurisdiction outside of Alberta, which is regularly ranked low in terms of health indicators, was invited to produce their own NRC but declined in fear of being singled out with low scores. There is the possibility for unintended consequences of weakening relationships, or deterring the potential for future engagement, if stakeholders feel that grades put their sector in a poor light. To address this challenge, it is important to work with stakeholders embedded in the community early on in the research process to assess whether alterations to the framework, such as a focus on strengths-based recommendations as opposed to grades, would be helpful for their community.

## Summary of lessons learned

The processes of developing, implementing and revising the NRC have provided important lessons applicable to the NRC’s target stakeholder groups: (i) researchers, (ii) practitioners, (iii) policymakers and (iv) the general public.

- *Researchers.* The exercise of identifying indicator areas in which it appears important to intervene, and collating the supporting evidence for each indicator, has identified important gaps in the evidence base that should be targeted in future studies. Specifically, the NRC identifies policy-relevant questions that require answers, which may help researchers to avoid generating ‘policy free evidence’ that they are often accused of producing^(33, p. 813)^. Evaluations of the effectiveness, and in particular, cost-effectiveness of policies to reduce socioeconomic inequalities in children’s food environments are especially important^(33)^. According to policymakers, one of the most convincing types of evidence concerns the financial ‘costs of action, or inaction’^(33, p. 812)^.
- *Practitioners.* The NRC offers practitioners a flexible tool for use in their daily work which can be adapted to their practice setting. For example, school health facilitators can collect data for school-related indicators within the NRC (e.g., healthy food availability in schools) and advocate for changes (e.g., school nutrition policy implementation) based on the results. Practitioners have a unique ability to make immediate changes in their practice. This was evident in the case of Alberta Health Services public health dietitians who have been able to use the NRC as a tool to assess local community food environments, with analytic support from our university-based research team.
- *Policymakers.* The NRC collates a broad array of evidence in an accessible, readable document, which can serve to amplify its impact^(34)^. Moreover, the media attention generated by the NRC release increases policymakers’ awareness of its existence. This awareness may in turn prompt action or, if the time is not right to act now, will provide readily available evidence to inform policy decisions at critical time points when policy windows open in the future^(27)^. Given the importance of timely action during such ‘window openings’, the NRC’s role in this respect should not be underestimated. If evidence-based policy is to become the norm, rather than the exception, stronger linkages will be needed between researchers and policymakers^(33)^. Additionally, the local data generated from the NRC may have more impact in decision-making processes affecting these jurisdictions than research that has been conducted elsewhere.
- *The media and general public.* The NRC can aid the media and general public in drawing policymakers’ attention to the issues that concern them, by providing a compelling evidence-based case for action packaged into a single, engaging document. Indeed, policymakers have highlighted how ‘a good story’ can influence politics^(33, p. 812)^.

## Future directions

The longitudinal impact of the NRC on policy readiness, environmental change, children’s diets and health outcomes will be described in future publications. Efforts are underway to institutionalise the NRC to ensure maximum benefits are derived from this work, a prospect that will require a stable source of long-term funding. While the NRC is currently intended for application at a provincial/territorial level in Canada, we aim to produce a national NRC in the future, once a critical mass of jurisdictions has adopted the NRC. Work on a national scale will enable benchmarking of food environments between jurisdictions. A number of Canadian provinces are currently establishing the infrastructure needed to produce their own provincial NRC. Our team has developed a ‘NRC toolkit’ (available upon request) to facilitate this process, and we plan to create an online hub with NRC resources.

## Conclusions

Over the past 5 years, the NRC has demonstrated its value as an engaging and accessible tool for benchmarking progress in improving children’s food environments in Alberta, Canada. The NRC framework, production process and knowledge translation strategy have been increasingly refined to enhance its relevance and utility for its target audiences of researchers, practitioners, policymakers and the general public. Partnerships, including those with the EWG, have been essential to annual NRC production and knowledge translation. Working alongside the EWG, such as through grading NRC indicators, has facilitated reflection on lessons learned from our successes and challenges in producing the NRC each year. Overall, the NRC may help to foster a supportive climate for policy adoption and implementation by stimulating dialogue about the importance of healthy food environments for children and youth. Efforts are in place to expand the NRC across Canada.
